# Copy Number Variations in a Population-Based Study of Charcot-Marie-Tooth Disease

**DOI:** 10.1155/2015/960404

**Published:** 2015-01-08

**Authors:** Helle Høyer, Geir J. Braathen, Anette K. Eek, Gry B. N. Nordang, Camilla F. Skjelbred, Michael B. Russell

**Affiliations:** ^1^Section of Medical Genetics, Department of Laboratory Medicine, Telemark Hospital, 3710 Skien, Norway; ^2^Head and Neck Research Group, Research Centre, Akershus University Hospital, 1478 Lørenskog, Norway; ^3^Campus Akershus University Hospital, University of Oslo, 1474 Nordbyhagen, Norway

## Abstract

Copy number variations (CNVs) are important in relation to diversity and evolution but can sometimes cause disease. The most common genetic cause of the inherited peripheral neuropathy Charcot-Marie-Tooth disease is the *PMP22* duplication; otherwise, CNVs have been considered rare. We investigated CNVs in a population-based sample of Charcot-Marie-Tooth (CMT) families. The 81 CMT families had previously been screened for the *PMP22* duplication and point mutations in 51 peripheral neuropathy genes, and a genetic cause was identified in 37 CMT families (46%). Index patients from the 44 CMT families with an unknown genetic diagnosis were analysed by whole-genome array comparative genomic hybridization to investigate the entire genome for larger CNVs and multiplex ligation-dependent probe amplification to detect smaller intragenomic CNVs in *MFN2* and *MPZ*. One patient had the pathogenic *PMP22* duplication not detected by previous methods. Three patients had potentially pathogenic CNVs in the *CNTNAP2*, *LAMA2*, or *SEMA5A*, that is, genes related to neuromuscular or neurodevelopmental disease. Genotype and phenotype correlation indicated likely pathogenicity for the *LAMA2* CNV, whereas the *CNTNAP2* and *SEMA5A* CNVs remained potentially pathogenic. Except the *PMP22* duplication, disease causing CNVs are rare but may cause CMT in about 1% (95% CI 0–7%) of the Norwegian CMT families.

## 1. Introduction

Copy number variations (CNVs) are a deletion or duplication of >1 kb genomic area [1]. CNVs have been identified as a key genetic determinant of evolution, diversity, and sometimes genetic disorders [1–3].

Charcot-Marie-Tooth (CMT) disease is a hereditary peripheral motor and sensory neuropathy. The most common single cause is a 1.4 Mb duplication of the* PMP22 *at 17p12, while a deletion of this gene causes hereditary neuropathy with pressure palsies (HNPP) [4–6]. Otherwise, CMT is most often caused by point mutations in one of the almost 50 identified CMT genes [7–10]. Non-*PMP22 *duplication CNVs have been considered rare. One study investigated CNVs in 34 peripheral neuropathy genes among 97 peripheral neuropathy patients and found no pathogenic CNVs [11]. CNVs have, however, been identified in* MFN2*,* MPZ*, and* NDRG1 *in single families with CMT disease [12–15]. In relation to point mutations the most frequent CMT genes have been investigated thoroughly in several populations, but only recently has next generation sequencing (NGS) technology made it feasible to sequence all known CMT genes [7, 16–19]. However, 54% of our Norwegian CMT families from the general population remained without a genetic diagnosis after exclusion of the* PMP22* duplication and the NGS sequencing of 33 CMT genes and 19 other neuropathy genes [19, 20]. Additional CMT genes have been identified after this study [7–10], and several genes are likely to be identified in future, but it could also be speculated that non-*PMP22* duplication CNVs might be relevant.

The aim of this study was to investigate the role of CNVs in Norwegian CMT families from the general population. Whole-genome array comparative genomic hybridization (array CGH) was applied in order to detect CNVs in all currently known CMT and related peripheral neuropathy genes (~90) and to investigate new candidate CMT genes. In addition multiplex ligation-dependent probe amplification (MLPA) was applied for detection of smaller intragenomic CNVs in* MFN2* and* MPZ*, since CNVs were already reported in three families [12, 14, 15].

## 2. Materials and Methods

### 2.1. Study Population

People with CMT residing in eastern Akershus County, Norway, were included in this population-based study; in total 245 affected persons from 116 CMT families were identified [20]. DNA was available in 81 CMT families, amounting to 189 affected individuals. The families had previously been tested for the PMP22 duplication by real-time quantitative PCR and point mutations in most known neuropathy genes [19, 20]. In addition, a duplication of MPZ was identified in one family [12]. In total, a genetic cause was identified in 46% of the population, 37/81 families. A comprehensive description of the study population has been published previously [20].

This study applied array CGH for whole-genome CNV detection and MLPA for detection of intragenomic CNVs in* MFN2* and* MPZ* on affected index patients from the remaining 44 CMT families without a genetic diagnosis. The genotyped patients were classified neurophysiologically as CMT1 (*n* = 17), CMT2 (*n* = 21), intermediate CMT (*n* = 1), or patients with an unknown neurophysiological phenotype (*n* = 5). Among these were also patients found to have variants of uncertain pathogenicity in previous publications [19]. In addition we included 16 index patients with known pathogenic point mutation from our population in the array CGH analysis. The Norwegian Regional Ethical Committee for Medical and Health Research Ethics approved the project, and the participants gave written informed consent.

### 2.2. CNVs Detection by Array CGH and MLPA

Array CGH was performed according to the protocol of the manufacturer, using 180 K (180000 probes) whole-genome arrays (Agilent Technologies, Inc., Santa Clara, USA). Each sample was labelled with Cy5 and each corresponding male or female reference with Cy3. The arrays were scanned on an Agilent G2505C DNA Microarray Scanner, and the CytoGenomics Software v. 2.5 was used for analysis of the data. A log2 Cy5/Cy3 ratio of ≥0.30 or ≤−0.40 among three adjacent probes was defined as gain or loss of genomic material, respectively, and was included in the further classification. Samples with derivative log ratio spread above <0.20 (excellent) were evaluated for reanalysis.

MLPA was performed according to the protocol of the manufacturer, using the MLPA kit P143 for the* MFN2 *and* MPZ* region (CMT1B/2A) (MRC Holland, Amsterdam, Netherlands). Data were analysed by the program GeneMarker v. 2.20 (SoftGenetics LLC, State College, PA, USA). Average peak areas of three different normal DNA samples were used as a reference.

### 2.3. Classification of Variants

The detected CNVs were analysed and classified based on criteria already present in our laboratory for CNV analysis and with a basis in published literature [18, 21, 22]. To aid the classification process, we used our in-house database that contains CNV data from 1140 patients and relatives, online benign and disease databases, such as DGV, ISCA, and DECHIPER [23–25], and reports in the HGMD, OMIM, and related literature [9, 26]. Patients with CMT are not routinely analysed by array CGH and thus are not included in our in-house database.

The classification criteria were as follows: (1) benign CNVs, CNVs present in ≥5 patients in our in-house database as well as being present in the DGV and ISCA benign databases; (2) probably benign CNVs, CNVs present in three to five patients in our in-house database or CNVs reported ≥3 in the DGV or ISCA benign database or CNVs in genes not related to the peripheral nervous system or CNVs in areas without known genes and not in close vicinity to other peripheral neuropathy genes; (3) potentially pathogenic CNVs, novel CNVs or CNVs observed ≤2 in our in-house database, DGV, or ISCA benign databases situated in genes associated with peripheral nerves, neuromuscular, or neurodevelopmental disorders; (4) pathogenic CNVs, CNVs known to cause peripheral neuropathies.

Patients with previously identified point mutations in recessive genes were also examined manually for CNVs in these genes [19].

Identified variants classified potentially as pathogenic were submitted to the ClinVar database (http://www.ncbi.nlm.nih.gov/clinvar/). Accession numbers are SCV000189426, SCV000189427, and SCV000189428.

## 3. Results

Among the 44 index patients we identified 588 CNVs by array CGH. The majority of CNVs were classified as benign (*n* = 493) or probably benign (*n* = 91). One duplication was classified as pathogenic, and one duplication and two deletions were classified as potentially pathogenic (Table 1). Two of the samples were reanalysed due to low quality.

No CNVs were detected in the* MFN2 *and* MPZ* genes after the MLPA analysis.

The CNV classified as pathogenic was the common 1.3 mega base* PMP22 *duplication. The duplication was verified by MLPA.

**Figure 1:**
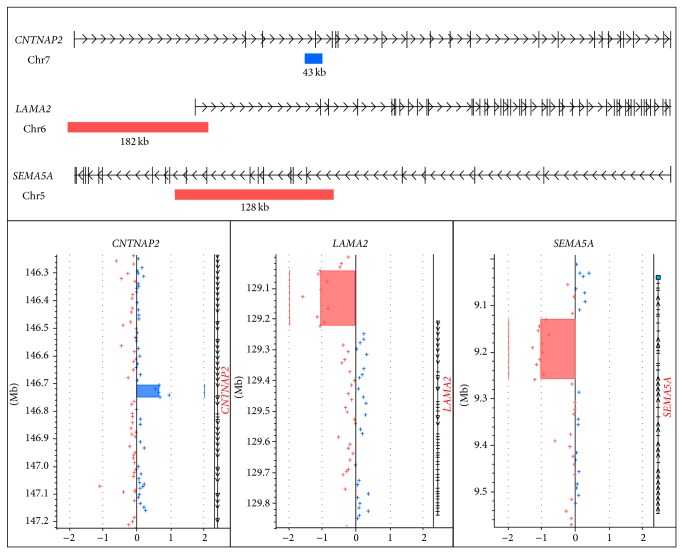
Illustration of potentially disease causing CNVs found in the genes* CNTNAP2*,* LAMA2,* and* SEMA5A*. The top part of the figure shows location and size of the CNVs in relation to corresponding RefSeq genes downloaded from the UCSC Genome Browser (http://genome.ucsc.edu/). The lower part of the figure shows the software plot. Blue bar indicates duplication, and red bar indicates deletion.

Three novel CNVs in the* CNTNAP2*,* LAMA2,* and* SEMA5A* were classified as potentially pathogenic (Table 1 and Figure 1). The CNVs in* CNTNAP2* and* SEMA5A* were intragenomic, while the CNV in* LAMA2* covered both promoter and exon 1. The index patient with the* CTNAP2* duplication had an affected sister with the same duplication. The* LAMA2* deletion was not detected in the unaffected mother and the* SEMA5A* deletion was not detected in the unaffected father.

## 4. Discussion

### 4.1. Main Findings

The main result of this study is that among 44 patients that had been previously screened negative for point mutations and small indels in 51 peripheral neuropathy genes only one CNV, a* PMP22* duplication, was detected in known neuropathy genes by whole-genome CNV analysis. However we detected novel potentially pathogenic CNVs among three patients in genes related to other neuromuscular or neurodevelopmental diseases. No CNVs were detected by MLPA analysis of the* MFN2 *and* MPZ *genes among the 44 patients.

### 4.2. CNVs and Neuromuscular Disorders

It is well established that CNVs play a major role in human variation and disease; in fact more base pairs vary between individuals due to CNVs compared with single nucleotide polymorphisms [1, 3]. Whole-genome CNV analysis is routinely performed in clinical genetic laboratories to detect aberrations in patients with syndromes [1, 2]. But CNVs are also important in relation to neurological disorders. The* PMP22* duplication is the most common cause of CMT; spinal muscle atrophy is mostly caused by deletions in the SMN1 gene and deletions of* SPAST* have been identified in hereditary spastic paraplegia [4, 6, 27–29]. In relation to peripheral neuropathy, CNVs other than the PMP22 duplication and deletion have been considered rare. No pathogenic CNVs were identified after investigating 97 peripheral neuropathy patients for CNVs in 34 neuropathy genes [11]. It has been speculated whether other rare CNVs could cause peripheral neuropathies after discovering 17 unique CNVs at the PMP22 locus in CMT patients [30]. But so far only two duplications of MPZ, one duplication of NDGR1, and one deletion of MFN2 have been identified in separate studies on single families [12–15].

### 4.3. Methodological Considerations

During the two last years, NGS has made possible a rapid increase in the number of CMT genes and several new CMT genes are expected to be identified in the future. The clinical and genetic overlap with other diseases of the nervous system, such as amyotrophic lateral sclerosis, ataxia, hereditary spastic paraplegia, and muscular dystrophy, has also become more evident [8, 31–33]. In a recent exome sequencing study of 27 peripheral neuropathy patients, the authors identified five certainly or potentially pathogenic point mutations in genes related to other neuromuscular or neurodegenerative disorders and three potentially pathogenic variants in other new candidate genes [18]. The application of whole-genome array CGH in this study makes it possible to detect CNVs not only in peripheral neuropathy genes but also in other genes related to other neuromuscular and neurodevelopmental disorders. To our knowledge this is the first study to apply whole-genome CNV analysis on a population of CMT patients. The disadvantage of applying whole-genome CNV analysis is the limitation in detection of smaller sized CNVs. Array CGH containing 180000 probes, as applied in this study, has an average limit of detection at 26000 bases. Thus, small intragenomic CNVs were missed. A custom array CGH analysis, targeting only selected genes, would be a possible solution to overcome this, but then other unknown genes would be missed. In the future it might be easier to detect both single nucleotide variants and CNVs by exome targeted next generation sequencing methods; several computational programs have been developed with different approaches, but they have not yet been thoroughly tested [34]. Array CGH is a well-established method which is regarded as robust and reliable [35].

### 4.4. Genotype-Phenotype Correlations

One patient carried the* PMP22* duplication, which was verified by MLPA. This patient had a demyelinating neuropathy corresponding to the* PMP22* phenotype. It is uncertain why this duplication was missed by real-time PCR, but it might be due to mix-up of samples prior to real-time PCR.

Three patients had novel CNVs in genes with both known function in the peripheral nervous system and association with other neuromuscular or neurodevelopmental disease phenotypes.

One patient and her affected sister had a duplication of exon 4 in the* CNTNAP2* gene. Age of onset was five and six years, and the phenotype was CMT2. The* CNTNAP2* encodes the protein Caspr2 which facilitates cell-cell interactions in the nervous system and is involved in the clustering of potassium channels at the juxtaparanode of myelinated axons [36–38]. Potassium channels are important in restoring the resting potential of nerve cells in the central and peripheral nervous system [36–38]. There are also evidences that Caspr2 has a role in the morphological formation synapses [39]. The* CNTNAP2* gene has been implicated in a broad range of phenotypes, including autism, Pitt-Hopkins-like mental retardation, schizophrenia, dyslexia and language impairment, delayed motor development, and mild ataxia [9, 36, 39]. The disruptions have been translocations, deletions, and nonsense or missense mutations. Most disruptions have been heterozygous mutations with disease burden placed in the area of exons 2–5, and the patients with homozygous mutations have been more severely affected [9, 36, 39]. Caspr2 is also involved in voltage-gated potassium channel autoimmunity, associated with phenotypes like neuromyotonia, Morvan's syndrome, limbic encephalitis, seizures, and frontotemporal dementia among others [40, 41]. Caspr2 autoantibodies have especially been associated with peripheral presentations and peripheral motor excitability. Recently two cases of Guillain-Barré syndrome were also reported [41, 42]. The diseases associated with* CNTNAP2* and its product Caspr2 are contradicting. Genetic causes have manifestation in the central nervous system often with language impairment [9, 36, 39], whereas autoantibodies against Caspr2 are mostly associated with a peripheral presentation. Given the extensive number of pleiotropic events of* CNTNAP2*, a role in CMT cannot be entirely excluded, although so far CMT has not been linked to* CNTNAP2*.

A patient with CMT2, 39-year-old at onset, had a deletion of the promoter and first 112 amino acids of* LAMA2*. As the translation start codon is deleted, it can be assumed that this allele is not expressed leading to partial* LAMA2* expression.* LAMA2* encodes the laminin *α*2 chain of the heterotrimeric laminin 2 protein (Merosin) found in the basement membranes of striated muscle and Schwann cells. In Schwann cells, laminin 2 is situated in the extracellular matrix attached to the membrane; the protein mediates Schwann cell-axon interaction and plays a critical role in myelination [43–45]. Mutations in* LAMA2* causing laminin *α*2 deficiency are widely associated with severe autosomal recessive congenital muscular dystrophy (CMD). Partial laminin *α*2 deficiency causes a milder CMD phenotype. CMD is characterized by severe muscle weakness, hypotonia, joint contractures, dysmyelinating peripheral neuropathy, and brain defects [43–46]. It is further believed that the peripheral neuropathy plays a critical role in the pathogenesis of CMD [43–45]. CMD patients show mild to moderate reduction of motor nerve conduction velocity (NCV), whereas sensory NCV is normal [47]. The same results are obtained from spontaneous mutant mice, whereas knock-out mice show severe reduction of motor NCV [43]. A recent research article reported a girl presenting with a sensimotor polyneuropathy with possible axonal involvement. Further examination revealed mildly dystrophic features and subtle laminin *α*2 deficiency. Molecular studies identified two heterozygous* LAMA2* mutations [48]. Our patient showed an axonal CMT phenotype with motor and sensory NCV in the same range as the previously reported girl [48]. Thus, we consider it quite probably that the* LAMA2* deletion observed in this patient leads to partial laminin *α*2 deficiency and is likely causative of the patient's peripheral neuropathy.

One patient with CMT1, 12-year-old at onset, had a novel deletion involving 442 highly conserved amino acids, out of a total of 1075, of the* SEMA5A* gene.* SEMA5A* belongs to a large class of proteins, the semaphorins, which are involved in the guidance of axons throughout the nervous system [49, 50]. Both a delay in motor axon extension and an aberrant branching of motor axons were observed in Zebrafishes, when the* SEMA5A* gene was knocked out, indicating a bifunctional role of* SEMA5A *[50]. Mice showed high neuronal* SEMA5A* expression during development but minor expression in adults [49]. Single nucleotide polymorphisms of the* SEMA5A* have been suspected in autism spectrum disorders and Parkinson's disease [51, 52].* SEMA5A* is also included in the Cri-du-chat syndrome deleted region and the protein product semaphorin 5A is significantly elevated in rheumatoid arthritis [53, 54]. Given the importance of* SEMA5A* in the guidance of axons in the central and peripheral nervous system and the pleiotropic events reported for this gene, a role in CMT cannot be entirely excluded.

Based on the genotype-phenotype correlations of these three CNVs, we consider* LAMA2* to be a new potential target gene for peripheral neuropathy, whereas* CNTNAP2* and* SEMA5A* remain potentially pathogenic. However, further functional studies are needed to confirm or disconfirm the pathogenicity of these CNVs.

In addition, one patient had a 1.3 mega base duplication at 16p13.11, which has been associated with a broad range of neurodevelopmental disorders, including autism, ADHD, intellectual disability, and schizophrenia in large cohorts. Due to reduced penetrance at this locus and no signs of peripheral neuropathy, this duplication was classified as probably benign in this context [55, 56].

The analysis of the additional 16 index patients with previous identified point mutations did not reveal any potentially pathogenic CNVs. However, two of the 16 patients with known point mutation had large CNVs classified as probably benign. One patient had a 1.5 mega base duplication at 22q11.21, and one patient had a duplication of the X chromosome. The phenotype of the 22q11.21 duplication is extremely variable, ranging from normal to learning disability to congenital defects but not associated with peripheral nerve disease [57]. Motor problems and impairments in coordination, balance, and strength had been reported for patients with Klinefelter syndrome (XXY), but that is unlikely to be confused with CMT [58].

## 5. Conclusion

The results of this study on a Norwegian population of CMT patients identified one pathogenic* PMP22* duplication that was not previously detected by real-time PCR and three potentially pathogenic CNVs, of which the deletion of* LAMA2* was considered to be likely pathogenic and the duplication of* CNTNAP2* and deletion of* SEMA5A* were considered to be potentially pathogenic. This study is in line with the results of the previous study by Huang et al.; copy number variations in CMT genes other than the* PMP22* duplication are a rare cause of CMT [11]. Apart from the duplication of* MPZ* discovered in this population in a previous study [12], other intragenomic CNVs in* MFN2* and* MPZ* also seem to be rare. In relation to these findings, we consider array CGH an unnecessary routine analysis in patients with CMT, but it could be beneficial in families without a genetic diagnosis after analysis of point mutations. For research purposes, CNVs should be further explored in larger cohorts to establish their relevance in relation to peripheral neuropathy.

## Figures and Tables

**Table 1:** Phenotype in CMT patients with pathogenic or potentially pathogenic CNVs.

Patient ID	CMT type	Age of onset	CNV type	Genomic coordinates (Hg19)	Size	Probes	Genes involved
Pathogenic CNVs							
0784	CMT1	40	Duplication	Chr17:14,100,118-15,442,066	1341 kb	68	*COX10, CDRT15, HS3ST3B1, MGC12916, CDRT7, PMP22, TEKT3, CDRT4, FAM18B2-CDRT4, FAM18B2 *

Potentially pathogenic CNVs							
0886	CMT2	6	Duplication	Chr7:146,705,271-146,748,724	43 kb	5	*CNTNAP2 *
0217	CMT2	39	Deletion	Chr6:129,040,519-129,222,690	182 kb	9	*LAMA2 *
0619	CMT1	12	Deletion	Chr5:9,129,755-9,257,708	128 kb	11	*SEMA5A *

Probably benign CNVs							
Total of 91 CNVs among all 44 patients							

Benign CNVs							
Total of 493 CNVs among all 44 patients							

CMT1 = demyelinating Charcot-Marie-Tooth; CMT2 = axonal Charcot-Marie-Tooth; hg = human genome build; kb = kilo bases.
